# Effects of Nitrogen and Phosphorus Limitation on Fatty Acid Contents in *Aspergillus oryzae*

**DOI:** 10.3389/fmicb.2021.739569

**Published:** 2021-10-21

**Authors:** Gongbo Lv, Ying Xu, Yayi Tu, Xiaojie Cheng, Bin Zeng, Jianhua Huang, Bin He

**Affiliations:** ^1^Jiangxi Key Laboratory of Bioprocess Engineering and Co-Innovation Center for In-Vitro Diagnostic Reagents and Devices of Jiangxi Province, College of Life Sciences, Jiangxi Science and Technology Normal University, Nanchang, China; ^2^College of Life Sciences, Sichuan Normal University, Chengdu, China; ^3^College of Pharmacy, Shenzhen Technology University, Shenzhen, China

**Keywords:** nitrogen and phosphorus limitation, fatty acid, *Aspergillus oryzae*, transcriptome, differentially expressed genes

## Abstract

*Aspergillus oryzae*, commonly known as koji mold, has been widely used for the large-scale production of food products (sake, makgeolli, and soy sauce) and can accumulate a high level of lipids. In the present study, we showed the dynamic changes in *A. oryzae* mycelium growth and conidia formation under nitrogen and phosphorus nutrient stress. The fatty acid profile of *A. oryzae* was determined and the content of unsaturated fatty acid was found increased under nitrogen and phosphorus limitation. Oleic acid (C_18:1_), linoleic acid (C_18:2_), and γ-linolenic acid (C_18:3_) production were increased on five nitrogen and phosphorus limitation media, especially on nitrogen deep limitation and phosphorus limitation group, showing a 1. 2–, 1. 6–, and 2.4-fold increment, respectively, compared with the control. Transcriptomic analysis showed the expression profile of genes related to nitrogen metabolism, citrate cycle, and linoleic acid synthesis, resulting in the accumulation of unsaturated fatty acid. qRT-PCR results further confirmed the reliability and availability of the differentially expressed genes obtained from the transcriptome analysis. Our study provides a global transcriptome characterization of the nitrogen and phosphorus nutrient stress adaptation process in *A. oryzae*. It also revealed that the molecular mechanisms of *A. oryzae* respond to nitrogen and phosphorus stress. Our finding facilitates the construction of industrial strains with a nutrient-limited tolerance.

## Introduction

Fatty acids (FAs) are carboxylic acids with long aliphatic chains, which may be straight or branched, saturated or unsaturated, and the majority of FAs generally found in nature have 6–18 carbon atoms (Mohan et al., 2015). FAs are comprised of saturated fatty acids (SFAs) and unsaturated fatty acids (UFAs). UFAs are typically biosynthesized by desaturation and are vulnerable to oxidation caused by hydration (water), oxidation (oxygen), metallic atoms, or microbes. UFAs can be divided into monounsaturated fatty acids and polyunsaturated fatty acids based on the number of carbon–carbon double bonds (Grama et al., 2014). UFAs are also a component of the phospholipids in cell membranes and contribute to maintaining membrane fluidity. Furthermore, it is a fundamental component in human and animal nutrition and with the function of blood lipid regulation, immune regulation, and retina maintenance for improving vision. Traditionally, UFAs are obtained from fish and vegetable oils. However, there are several limitations of fish oils as a source of essential UFA supply, such as the presence of teratogen, carcinogen, mutagen, and non-carcinogen contaminants, including dioxin-like polychlorinated biphenyls, methyl mercury, heavy metals (Hg, Cr, Pb, and As), and antibiotics (Abedi and Sahari, 2014).

Considerable researches that documented the improvement of FA content via oleaginous microorganisms including bacteria, microalgae, yeasts, and filamentous fungi, which possess strong secretion ability has attracted great attention in recent years (Ramanaiah et al., 2007). Microalgae is a high-lipid microbe, with the characteristic of high photosynthetic efficiency, short life cycle, and easy scale-up, which has made it a potential biodiesel feedstock (Bharathiraja et al., 2015). Moreover, most microalgae biomasses are a rich source of ω-3 and ω-6 FAs, essential amino acids, and the FA profile is mainly C_16_ and C_18_, containing oleic, palmitic, linoleic, and stearic acid (Koutinas et al., 2014). Contrary to microalgae, which need larger acreages to culture algae and long fermentation period, bacteria grow extremely rapidly to reach huge biomass, and its culture method is relatively easy. Several storage-lipid-accumulating bacteria, such as the actinomycete group, are capable of synthesizing a large number of FAs (over 70% of the cellular dry weight) under growth-restricted conditions and then accumulate them intracellularly as triacylglycerols (Alvarez et al., 2002). Besides, oleaginous microorganisms, filamentous fungi and yeasts have been exploited to be a promising alternative for the production of FAs, for example, *Mortierella alpina*, *Fusarium solani*, and *Rhodotorula glutinis* (Takeno et al., 2005; Rasmey et al., 2020). Analogous to microalgae, oleaginous yeasts and molds accumulate triacylglycerols mainly in UFAs, C_16_, and C_18_. The previous study has reported several yeast strains, such as *Rhodosporidium* sp., *Yarrowia*, *Rhodotorula* sp., and *Lipomyces* sp. are capable of synthesizing and accumulating intracellular lipids as high as 70–75% of their biomass dry weight (Athenaki et al., 2018). In particular, the oleaginous yeast *Cryptococcus curvatus* can accumulate storage lipid over 60% on a dry weight basis, while under N-limiting conditions, these lipids usually consist of 44% SFAs, which is similar to many plant seed oils (Meng et al., 2009). Extensive number of researches has been carried out on filamentous fungi to improve the content of FAs and reduce the FA production costs, such as *Mortierella alpina*, *Mucor circinelloides*, *Lichtheimia corymbifera*, *Aspergillus oryzae*, and *Trichoderma viride* NRC 314 (Ali et al., 2017; Dzurendova et al., 2020b). The main advantages of these microorganisms include fast growth, no need for light energy, easy scalability, and the ability to utilize a broad spectrum of low-cost substrates, such as whey, molasses, sugar beet pulp, corncob waste liquor, and other industrial and agro-industrial byproducts (Economou et al., 2011; Ozsoya et al., 2015; Ali et al., 2017).

To improve the economic competitiveness of microbial lipids compared with plant- and animal-derived oils, the unremitting efforts made and continuous research and development of the oleaginous oil production of microorganisms were carried out over the past several years. Four main strategies are mentioned as follows to maximize the production of microorganism lipids: screening for potential oleaginous microorganisms, and genetic, metabolic, and morphology engineering. Several engineering approaches have resulted in the development of strains with enhanced FA production capabilities. Ali and El-Ghonemy have reported that a large amount of palmitoleic acid (C_16__:__1_), linoleic acid, and linolenic acid in the biomass of *Trichoderma viride* NRC 314, up to 30, 23, and 13% of total fatty acids (TFAs), respectively, over other fungal strains (Thanaa and Ali, 2014). Later, they screened an oleaginous fungus among 13 filamentous fungi under the same growth conditions, *Penicillium brevicompactum* NRC 829, which gave the highest amount of lipid accumulation. The optimum concentrations of the most significant medium components (different carbon and nitrogen sources, as well as various metal salts) required to improve the lipid production by *Penicillium brevicompactum* were also obtained (Ali et al., 2017). Kosa et al. (2018) used the Duetz microtiter plate system combined with Fourier transform infrared spectroscopy for high-throughput screening of the potential of 100 Mucoromycota fungi including *Amylomyces*, *Mucor*, and *Mortierella* species, for their ability to produce low- and high-value lipids. Specifically, *Mucor fragilis* UBOCC-A-109196 and *Cunninghamella echinulata* VKM F-470 showed the highest gamma-linolenic acid content (up to 24.5 and 24.0% of TFAs, respectively), followed by alpha-linolenic acid and arachidonic acid (Kosa et al., 2018). As for genetic and metabolic engineering, two main pathways are mentioned as follows, the overexpression and disruption of the vital gene in lipid biosynthesis pathways, such as the fatty acid synthase and acyl-CoA synthetase gene. Likewise, there are also successful examples. In Ruenwai et al. (2009) reported that the overexpression of the acetyl-CoA carboxylase gene in a *Hansenula polymorpha* resulted in a 1.4-fold increase in FA content. Tamano et al. (2013) replaced the original promoters with the *tef1* promoters in *A. oryzae* and successfully increased the expression of the fatty acid synthase genes and the content of FAs and triglycerides by 2.4-fold. Later, in the reverse direction, by disruption of a predicted acyl-CoA synthetase gene AO090011000642, a 9.2-fold higher accumulation of intracellular FAs compared with the wild-type strain was also achieved (Tamano et al., 2015). Recently, Wong et al. (2021) employed a comprehensive analysis strategy to clarify the crucial pathways for free fatty acid secretory productivity and selected 16 biosynthesis genes for gene modification. Of them, overexpression of the fatty acid synthase gene resulted in a 2.1-fold increase in free FA productivity, and the disruption of the acyl-CoA synthetase gene resulted in a 9.2-fold increment (Wong et al., 2021). Besides, it has been reported that the lipid content of *A. oryzae* could be enhanced by the morphology engineering approach. The disruption of genes encoding α-1, 3-glucan synthase 1 resulted in lipid production about titer and productivity, which was significantly improved, compared with the wild type (Jeennor et al., 2019). Indeed, the extensive research achieved great improvement on FA content in the past decades, and stability of engineered strains and methods for obtaining stable production in industrial microbial processes are known to be key issues (Zhang et al., 1996).

Noteworthy, nutrient stress is an effective and promising strategy previously employed to improve UFA content in oleaginous microorganisms. Enormous efforts have been invested in diversified nutrients, such as nitrogen, carbon, phosphorus, and microelements, which are the essential nutrients involved in biomass growth and lipid accumulation in oleaginous microorganisms (Ratledge and Wynn, 2002). Nitrogen is required for the organism to synthesize amino acids, proteins, nucleic acids, enzymes, vitamins, alkaloids, and chlorophyll (Hajong et al., 2013). Furthermore, microorganisms have distinct abilities to use nitrogen by various assimilatory and dissimilatory pathways, and nitrogen is pivotal for the proliferation and growth of fungal cells and responsible for the creation of the backbone for FAs (akpınar, 2014; Milkessa, 2018). Among sources for nitrogen, ammonium is preferred, but nitrate can also be utilized by many filamentous fungi. In addition, several organic nitrogen sources have a good buffering system, whereas inorganic nitrogen sources, such as ammonia, excessively decrease the culture pH during NH_4_^+^ absorption, and thus, influence the fermentation and growth of filamentous fungi. Carbon is converted into triacylglycerides, which are stored in lipid bodies under the nitrogen-limiting condition. Phosphorus, usually taken up in inorganic form, is a key constituent of nucleic acids, and a part of phosphorylated molecules fundamental in lipid biosynthesis, such as reduced nicotinamide adenine dinucleotide phosphate (NADPH) and adenosine mono-, di-, tri-phosphate (AMP, ADP, ATP). Both of them are directly involved in FA synthesis, as reductant and energy transfer molecules, separately (Dzurendova et al., 2020b). Besides phosphorus being responsible for the formation of lipid droplets, it is part of the phospholipids of the lipid droplet membrane (Ratledge, 2004).

Culturing oleaginous microorganisms under the condition of these nutrients are sufficient or limited and are the main part of nutrient stress strategies for the increment of FA production. In yeast strains, UFAs production has also been studied for over 80 years. Athenaki et al. (2018) comprehensively reviewed the characteristic results of lipid production associated with the growth of oleaginous fungi and yeast on hydrophilic carbon sources. The content of lipid varies in different strains on distinct carbon sources and the same strain on different carbon sources under various cultivation configurations (Athenaki et al., 2018). Analogously, FA composition and metabolic activity changes of *Crypthecodinium cohnii* under different nitrogen sources and concentrations were significant. Among them, NaNO_3_ supported significantly higher lipid content, and the maximum level of C_16_–C_18_ content (% TFAs) was attained at the lipid accumulation stage under medium nitrogen treatments (Safdar et al., 2017). Nitrogen and phosphorus are directly involved in FA synthesis; thus, optimizing the ratio and concentration of nitrogen and phosphorus on the oleaginous microorganism medium is of great importance. Manipulation of culture conditions alters lipid content and FA profiles as reported by Sitepu et al. (2013), such as nitrogen starvation boosted accumulation of linoleic acid in many yeast strains. Moreover, previous studies have documented that nutrient stress can change the level of long-chain polyunsaturated fatty acids (LC-PUFAs) in *Porphyridium cruentum*. For example, Su et al. (2016) have shown that phosphate limitation promotes the linoleic acid proportion, while it decreases the proportions of arachidonic acid and eicosopentaenoic (C_20__:__5_), both of which have essential functions in the prevention and treatment of the brain or cardiovascular disease. Similarly, Hu et al. (2018) systematically reported the optimal conditions for the production of linoleic acid, and arachidonic acid, eicosapentaenoic acid, and TFA production in *Porphyridium cruentum*, nitrogen sufficiency and phosphorus deprivation, nitrogen sufficiency, and phosphorus limitation, respectively. Additionally, several exploitations have successfully increased the content and proportions of FAs (linoleic acid, eicosapentaenoic acid, etc.) by adopting distinct concentrations and ratios of nitrogen and phosphorus, such as green microalga *Dunaliella tertiolecta*, *Isochrysis zhangjiangensis*, and *Porphyridium cruentum* (Liang et al., 2017; Hu et al., 2018; Yu et al., 2019). Except the microalga and yeast strains, nitrogen and phosphorus stress have been studied in filamentous fungi as well. In a research of the nutrient-induced co-production of high-value metabolites in oleaginous *Mucoromycota* fungi, the lipid and other metabolite content varied in six different phosphorus levels and two nitrogen sources (Dzurendova et al., 2020a). Concomitantly, one lipid high-yielding strain with the potential for industrial application was screened, *Umbelopsis vinacea*, due to the observation of the highest lipid accumulation among all the tested strains. This result was validated by the following research on the influence of phosphorus sources and the nature of nitrogen substrate on lipid accumulation (Dzurendova et al., 2020b). Moreover, Kieliszek and Dourou (2021) have examined the effect of Se on the FA profile of total lipids and lipogenesis, after its inclusion as a component in the growth medium on model oleaginous microorganisms *Yarrowia lipolytica*. The results have shown that Se negatively affected both cell mass production and total lipid accumulation. Dynamic changes in the content of linoleic acid, palmitic (C_16__:__0_), and stearic acids in the ALE_70 and ACA DC 50109 strain revealed that Se seems to influence the activity or the expression of saturases, desaturases, and elongase in both strains.

Koji is cooked rice or soya beans that have been inoculated with a fermentation culture, koji mold (Machida et al., 2008). *A. oryzae*, commonly known as koji mold, is a safe production filamentous fungus identified by the FDA and WHO, which has been used in the manufacturing of oriental fermented foods and fungal secondary metabolites for thousands of years (Oikawa, 2020). Utilized as the starter of koji fermentation, *A. oryzae* is widely used as the starter in the preparation of koji in East Asia traditional fermentation industry involving the production of mirin, douche (fermented and salted black soybean), bean curd seasoning, vinegar, miso (soy bean paste), and shochu (distilled spirits) (Zhong et al., 2018; Daba et al., 2021). Additionally, *A. oryzae* has a prominent potential of secreting various hydrolytic enzymes (amylase, protease, etc.) to break down the bigger molecules such as starches, carbohydrates, proteins, and fats, and they are being reduced into smaller monosaccharides, amino acids, fatty acid chains, etc (Zhong et al., 2018). These enzymes are critical in the fermentation process, allowing prolonged degradation of biomass to energize subsequent fermentation and contribute to the characteristic color, flavor, and aroma of the fermented foods (Kitamoto, 2002; Feng et al., 2013). Moreover, *A. oryzae* has also been used in the enzyme industry as a cell factory for the production of numerous native and heterologous enzymes (Jin et al., 2021).

Until now, the molecular mechanisms of *A. oryzae* metabolism and related regulations during koji fermentation remain only partially understood, inhibiting targeted strain improvement, and effective strain maintenance against industrial strain degeneration. Therefore, precise physiological knowledge of *A. oryzae* metabolism and regulations is needed. Compared with the detailed experimental evidence for oleaginous microorganism nutrient demands for improving the content of UFAs, such as carbon, nitrogen, and phosphorus, there is still no consistent theoretical framework for the relationship between *A. oryzae* nutrient limitations or responses and FA biosynthesis. To elucidate the adaptive mechanisms of *A. oryzae* toward nutrient stress to improve the nutrient stress tolerance of *A. oryzae* strains, obtain highly stable engineered strains support for the industrial production and application, and methods that can maintain stable FA production in industrial microbial processes, here, we presented a case study of the effect of nutrient stress on FA content in oleaginous filamentous fungi *A. oryzae*.

## Materials and Methods

### Strain and Cultivation Conditions

*Aspergillus oryzae* strain 3.042 (CICC 40092) was obtained from the China Center of Industry Culture Collection and used to check the response to nitrogen (N) and phosphorus (P) limitation. The basal Czapek–Dox (CD) medium (3 g of NaNO_3_, 1 g of KH_2_PO_4_, 0.5 g of MgSO_4_, 0.5 g of KCl, 0.01 g of FeSO_4_, 30 g of sucrose, 20 g of agar powder, and sterile water up to 1,000 ml, pH 7.3 ± 0.2, autoclaved at 121°C for 15 min before use) was used. First of all, *A. oryzae* strain was pre-cultivated on a CD plate at 30°C for 3 days. According to the previous methods, spores were then harvested after 3 days of culture for follow-up experiments, and the conidia concentration was determined by using a hemocytometer (He et al., 2018a,b).

### Experimental Setup

Seven media recipes were used in this study, including both N and P sufficiency (control check, CK), N limitation and P sufficiency (N-), N deep limitation and P sufficiency (N- -), N sufficiency and P limitation (P-), both N and P limitation (N-P-), N deep limitation and P limitation (N- -P-), and both N and P deprivation (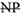). All of these media were based on the CD medium, and the basal CD culture medium was used as the CK. Sodium nitrate and potassium dihydrogen phosphate were used for N and P limitation, and their initial concentrations in all media are shown in Table 1. Then, a freshly prepared suspension with 1 × 10^7^ conidia was inoculated and cultured on distinct treatment plates covered by cellophane fresh at 30°C for 3 days. Each treatment was performed in three groups for the determination of biomass, spore density, and extraction of intracellular fatty acid. At least three replicates were carried out for each nutrient-limited group. It was followed by scraping, and then the fungal mycelia was dried overnight at 60°C for the biomass determination, and the density of spores was determined by a hemocytometer.

**TABLE 1:** The initial concentrations of sodium nitrate and potassium dihydrogen phosphate in all media (g/L).

Concentrations (g/L)	CK	N-	N- -	P-	N-P-	N- -P-	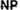
NaNO_3_	3	2	1	3	2	1	0
KH_2_PO_4_	1	1	1	0.5	0.5	0.5	0

### Extraction of Intracellular Fatty Acid From *Aspergillus oryzae*

Total lipids were extracted from whole-cell homogenates and methylated by following previously described approaches (He et al., 2018b). Briefly, *A. oryzae* mycelia were collected, washed with distilled water, and freeze dried. After that, the mycelia of each group were powdered and weighed. The same weight of mycelia powder was subjected to lipid extraction. The lipid extracts were incubated in chloroform with 2% H_2_SO_4_–MeOH solution at 70°C for 2 h to obtain fatty acid methyl esters (FAMEs). FAME components were separated and analyzed using coupled QP2010 gas chromatography-mass spectrometry (GC-MS) (Shimadzu, Kyoto, Japan). The system was equipped with Supelco SP-2340 fused silica capillary column (30 m × 0.25 mm i.d., with film thickness of 0.2 μm; Bellefonte, PA, United States). FAMEs were identified by comparing their mass spectra with a spectrum database. FA peaks were identified based on comparisons of their retention times to the external standards or similarity search. The relative amounts of individual FA components were performed by using the peak area of the most intensive ion of each peak, and the percentage of UFAs was also calculated (Kim and Salzberg, 2011; He et al., 2018b).

### Preparation of cDNA Libraries and RNA Sequencing

After scraping the cell samples, the total RNA extraction was performed with a fungal RNA kit (Omega Bio-tek, Norcross, GA, United States) according to the protocol of the manufacturer, coupled with DNA digestion. NanoDrop ND-1000 spectrophotometer (Thermo Fisher Scientific, Wilmington, DE, United States) and Bioanalyzer 2100 (Agilent Technologies, Palo Alto, CA, United States) were employed to analyze RNA concentration and integrity. To ensure reliability and reproducibility, equal quantities of RNA of each pool from three individual cultures were used for cDNA library construction. Thereafter, mRNA was enriched by Oligo(dT) beads. The enriched mRNA was then fragmented into several short fragments by fragmentation buffer at 94°C for 5 min and then reversely transcripted into cDNA using random primers. Using these mRNA fragments as templates, the first-strand and second-strand cDNA were synthesized through our previously described methods (He et al., 2018b). The cDNA fragments were purified using QIAquick PCR extraction kit, end repaired, and ligated to Illumina sequencing adapters to create the cDNA library. The size of the ligation products was selected by agarose gel electrophoresis, amplified, and sequenced using Illumina HiSeqTM 2500 (Biomarker Biotechnology Co., Beijing, China). The transcriptome datasets are raw reads containing adapters or low-quality bases. Therefore, reads will be further filtered by our previous criterion to get clean reads, and thus, removed (He et al., 2018a). Moreover, the Bowtie2 software (Bowtie 2: fast and sensitive read alignment)^1^ was used to remove reads that mapped to ribosome RNA (rRNA) database to get the final clean reads, which were further employed for assembly and transcriptome analysis (Langmead and Salzberg, 2012). RNA-Seq data of *A. oryzae* under nitrogen and phosphorus limitation were deposited in the NCBI/SRA database^2^, under the BioProject accession number PRJNA748192; BioSample: SAMN20309416-SAMN20309419.

### Transcriptome Analysis

The obtained clean-read datasets were aligned to *A. oryzae* 3.042 reference genome using Tophats2 (v2.0.3.12) (Kim and Salzberg, 2011). The RSEM software was used to quantify gene abundances, and the quantification of gene expression level was normalized using the FPKM (Fragments Per Kilobase of transcript per Million mapped reads) method (Mortazavi et al., 2008; Dewey and Bo, 2011). Differentially expressed genes (DEGs) across samples were identified using DESeq2 v1.18.1 on R package (version 3.4.2) (Love et al., 2014). The log2 (fold change) over 1 and false discovery rate (FDR) within 0.05 were set as the threshold for significant DEGs (He et al., 2018b). Then the identified DEGs were carried out into hierarchical clustering, with the KEGG pathway enrichment analysis.

### Quantitative Real-Time PCR Analysis

To validate the transcriptional level results from RNA-Seq data analysis, four genes including D9D1, D9D2, D12D1, and D12D2, which are involved in the linoleic acid biosynthesis in *A. oryzae*, were selected for real-time RT-PCR validation. Total RNA was extracted with E.Z.N.A. Fungal RNA Kit (Omega Bio-tek, Norcross, GA, United States) according to the protocols of the manufacturer. Reverse transcription of each RNA sample was performed to get the first-strand cDNA using the PrimeScript RT reagent Kit with gDNA Eraser (TaKaRa, Dalian, China). Real-time RT-PCR was performed using Real-Time PCR System (Bio-Rad). GAPDH served as the reference gene for normalization of the target gene expression and to correct for variation between samples. The thermal cycle for RT-PCR was as follows: 95°C for 2 min, followed by 40 cycles of 95°C for 10 s, 60°C for 15 s, and 72°C for 20 s. Melting curve analyses of the amplification products were performed at the end of each PCR reaction to ensure that only specific products were amplified. Primers used for the candidate genes are designed according to the Illumina sequencing data by using Primer Premier 5 and listed in Supplementary Table 1. The comparative 2^–ΔΔCT^ method was employed to calculate the relative expression between the target genes.

### Data Analysis

Three independent experiments were performed, and all of the data in this study are presented as mean ± SE of three replicates. Data from the same period were evaluated by one-way nested analysis of variance (ANOVA), followed by the least significant difference test (LSD) for mean comparison. All statistical analysis was performed with SAS 9.20 software (SAS Institute Inc., Cary, NC, United States) at *p* < 0.05. Moreover, all the difference analysis performed in this study was targeted between the control and nutrient-treated groups.

## Results

### Effects of Nitrogen and Phosphorus Limitation on Cell Growth of *Aspergillus oryzae*

**FIGURE 1:**
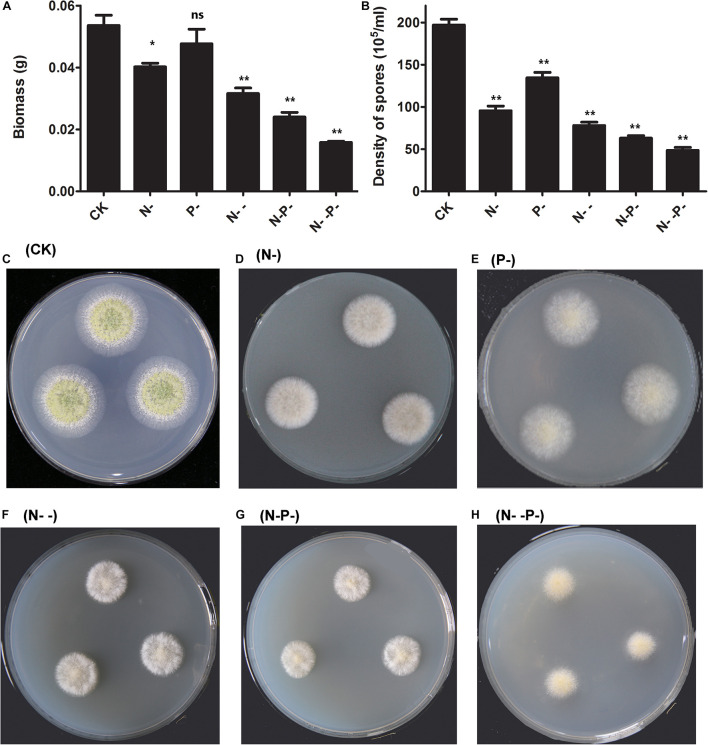
The dry biomass and density of spores as affected by nitrogen and phosphorus. **(A)** The dry biomass under nitrogen and phosphorus limitation and the control. The mycelia were collected by peeling them off from the plates and dried overnight for the determination of biomass. **(B)** The density of spores under nitrogen and phosphorus limitation and the control. **(C–H)** The phenotype of *A. oryzae* strain under the condition of control and distinct N/P limitation. CK, N-, P-, N- -, N- P-, and N- -P- represent the control and distinct N/P-limited groups, respectively. The bars represent the average (±SE) of biological repeats. Asterisks indicate statistically significant differences between groups (Student’s *t*-test): *p < 0.05, **p < 0.01, ns, no significant difference.

To investigate the effects of nitrogen and phosphorus limitation on cell growth of *A. oryzae*, a preliminary study of different concentrations of nitrogen and phosphorus treatments was conducted. After incubation for 48 h, the dry biomass of *A. oryzae* mycelioid colonies of the nitrogen and phosphorus-treated group were different from the control (Figure 1A). The dry biomass of *A. oryzae* on P- medium over that of N- and N- - medium was not much different from CK. The dry biomass detected on the N- -P- medium is the least. Additionally, the dried biomass end product of the control and distinct nitrogen/phosphorus-limited groups (g/g, fungal water content) is present in Supplementary Table 1. Based on the results obtained, no significant difference was observed in the fungal water content of the six groups, illustrating that nitrogen and phosphorus limitation has little effect on strain water content. It was noteworthy that the *A. oryzae* strain was not growing on the 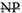 group, and thus, this group was excluded from the follow-up analysis. Overall, nitrogen and phosphorus limitation inhibited the dry biomass of *A. oryzae* to varying degrees, and thus, repressed cell growth. The density of spores of *A. oryzae* showed significant differences between the nitrogen and phosphorus limitation and the control (Figure 1B). Consistent with the result in dry biomass, the density of spores of *A. oryzae* on P- medium was less than CK, but superior to other media. Moreover, the density of spore decline on N-, N- -, N- P-, and N- -P- media sequentially indicated that the effect of inhibition increased with nitrogen and phosphorus limitation on these media. The phenotype of *A. oryzae* strain under the condition of control and distinct N/P limitation is present in Figures 1C–H. Notably, the diameter of *A. oryzae* strain in nitrogen/phosphorus-limited groups was less than that of the control, and it decreased with the decrease in nitrogen concentration. This phenomenon was in accordance with the change of the dry biomass and density of spores in *A. oryzae* strain. Furthermore, the conidia formation of *A. oryzae* on the P- group was inhibited but slightly better than that of other nutrient-limited groups. These results illustrated that nitrogen restriction has more significant effects on the growth, development, and conidia formation of *A. oryzae* strains compared with phosphorus limitation.

### Analysis of Fatty Acid Contents Under Nitrogen and Phosphorus Limitation of *Aspergillus oryzae*

The relationship between the degree of FA unsaturation of membrane lipids and nitrogen and phosphorus nutrient stress has been extensively studied on oleaginous fungi. However, the unsaturated mechanisms of *A. oryzae* FA against nutrient stress are poorly understood. In the present study, the FA content profiles of *A. oryzae* grown under nitrogen and phosphorus limitation and control were quantified by gas chromatography. Our results revealed that the contents of 18- and 16-carbon chain length FAs accounted for over 95.4% of TFAs, and the predominant were oleic acid, linoleic acid, stearic acid (C_18__:__0_), and palmitic acid (Table 2). When subjected to diverse nitrogen and phosphorus limitations, the contents of palmitic acid, stearic acid, arachidic acid (C_20__:__0_), and 2-decyltetradecanoic acid (C_24__:__0_) were decreased in varying degrees. Intriguingly, all these four FA contents on P- medium surpassed another four media and less than the control. This result showed that the effect of inhibition on FA contents caused by single phosphorus limitation is weaker than single nitrogen and other coupled limitation. Contrary to four FAs, the contents of oleic acid, linoleic acid, and γ-linolenic acid were increased on five nitrogen and phosphorus limitation media compared with the control. Especially on nitrogen deep limitation and phosphorus limitation group, the productions of these three UFAs were increased by 1. 2–, 1. 6–, and 2.4-fold, respectively, compared with the control. In addition, it is worth noting that the contents of these three FAs on P-medium are below other media. Additionally, a major change was observed in the degree of fatty acid unsaturation (Supplementary Figure 1). The intracellular content of UFAs increased by the largest 18% in adaption to nitrogen and phosphorus limitations. Among them, the intracellular content of UFAs was very close between the controls and P-medium, suggesting that a single phosphorus limitation might have little effect on the content of UFAs.

**TABLE 2:** Effects of nitrogen and phosphorus limitation on main FA composition and percentage of *Aspergillus oryzae*.

**Compositions (percentage of FA)**	**CK**	**N-**	**P-**	**N- -**	**N-P-**	**N- -P-**
C16:0	21.88	21.28	21.39	21.11	20.49	18.93
C18:0	25.73	23.00	25.36	16.67	16.34	13.16
C18:1n9	23.89	24.7	24.43	25.47	26.21	27.87
C18:2n6	20.06	23.06	21.09	28.98	29.18	32.38
C18:3n6	0.54	0.97	0.87	1.02	1.11	1.27
C20:0	2.04	1.16	1.17	1.11	1.01	0.95
C24:0	2.30	1.76	1.83	1.73	1.73	1.71

### Transcriptome Overview

To investigate the environmental stress response of *A. oryzae* induced by nitrogen and phosphorus, a transcriptome analysis based on RNA sequencing of *A. oryzae* that was subjected to various nitrogen and phosphorus limitations were performed. We chose three treated groups and the control for follow-up analysis due to their significant differences. This resulted into the generation of 46.36, 42.07, 44.56, and 41.01 million clean reads per library, respectively (Table 3). The GC content for all treatments was approximately 52%, and the % ≥ Q30 (99.9% accuracy of bases) was greater than 92% for all samples, indicating a good quality of the sequencing data. Also, the clean reads were aligned to *A. oryzae* 3042 genome sequence, and more than 78% of the clean reads for each sample were uniquely mapped to the genome. A summary of the RNA-seq sequencing is shown in Table 3.

**TABLE 3:** Summary of the sequencing data of *A. oryzae* under nitrogen and phosphorus limitation.

**Samples**	**Control**	**N-**	**P-**	**N-P-**
Clean reads	46,365,266	42,076,808	44,564,254	41,014,098
GC content	52.99%	52.12%	52.85%	52.40%
% ≥ Q30	93.18%	93.22%	92.72%	92.98%
Unique mapped	37,155,318 (80.14%)	34,049,299 (80.92%)	35,037,129 (78.62%)	32,419,457 (79.04%)
Multiple mapped	148,911 (0.32%)	116,274 (0.28%)	107,169 (0.24%)	90,981 (0.22%)

### Differentially Expressed Gene Analysis of *Aspergillus oryzae* Transcriptome Under Nitrogen and Phosphorus Limitation

**FIGURE 2:**
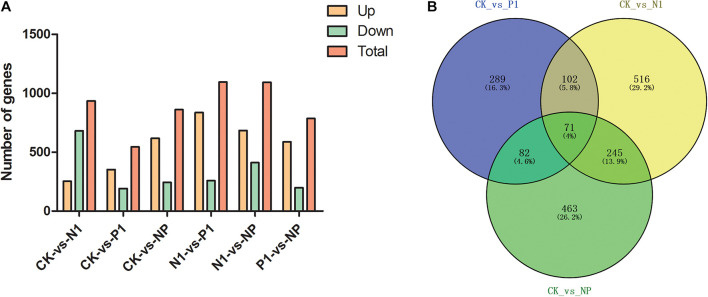
Distribution of differentially expressed genes (DEGs) in the six samples. **(A)** DEG distribution between control and nitrogen and phosphorus limitation. **(B)** Venn diagram exhibiting DEG distribution in three samples. CK, the control; P1, the single P limitation (P-); N1, the single N limitation (N-); NP, N deep limitation and P limitation (N- -P-).

To identify genes with altered expression levels with nitrogen and phosphorus limitation, we quantified the overall transcription levels of genes by RPKM metrics. The number of DEGs for CK-vs-N1, CK-vs-P1, CK-vs-NP, N1-vs-P1, N1-vs-NP, and P1-vs-NP groups [N1, the single N limitation (N-); P1, the single P limitation (P-); NP, N deep limitation and P limitation (N- -P-)], were 934, 544, 861, 1,094, 1,093, and 785, respectively (Figure 2A). There were 253 upregulated genes (27%) and 681 downregulated genes (73%) on control and N-medium, whereas the numbers of upregulated genes surpassed the downregulated genes on all remaining media. To better present the effect of N and P limitation on *A. oryzae*, four groups were selected for follow-up analysis due to their significant differences. A Venn diagram of DEG distribution was constructed (Figure 2B). It was observed that only 71 DEGs were commonly shared among the distinct condition of nitrogen and phosphorus limitations; the other commonly shared DEGs between every two groups were 102, 82, and 245, respectively. The numbers of specific DEGs between single nitrogen limitation and the control (516) were greater than that of the other two groups, indicating the involvement of complex developmental events on *A. oryzae* under single nitrogen limitation. Moreover, different common DEGs were found between CK-vs-N1, CK-vs-P1, and CK-vs-NP groups, suggesting that the response of *A. oryzae* to nitrogen and phosphorus limitation may be different.

### Differentially Expressed Genes Involved in General Metabolic Pathways

**FIGURE 3:**
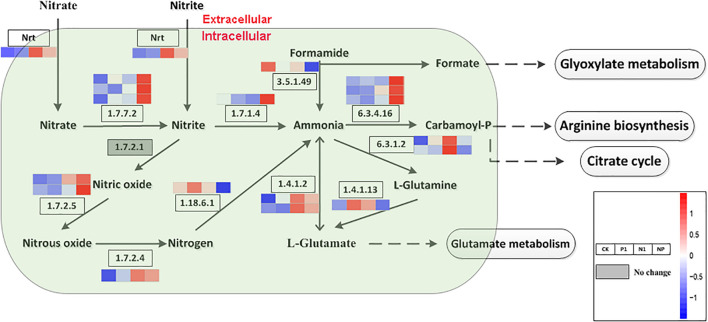
Key enzymes encoded by the DEGs involved in the nitrogen metabolism are enriched by KEGG analysis. CK, the control; P1, the single P limitation (P-); N1, the single N limitation (N-); NP, N deep limitation and P limitation (N- -P-).

Based on the KEGG enrichment analysis, nitrogen metabolism and citrate cycle (TCA cycle, Krebs cycle) is a general metabolic pathway involved in FA formation. A total of 18 DEGs encoding 11 enzymes were identified for the different steps in nitrogen metabolism. There were 3, 3, 2, 2, and 2 genes encoded by acetylcholinesterase (EC 1.7.7.2), carbamoyl-phosphate synthase (EC 6.3.4.16), glucokinase (EC 1.7.2.5), glutamate dehydrogenase (EC 1.4.1.2), and glutamine synthetase (EC 6.3.1.2), respectively; all of the other enzymes are encoded by single genes (Figure 3). Apart from the gene expression level on the pathway of formamine and nitrogen to ammonia (EC 3.5.1.49, EC 1.18.6.1), L-glutamine to L-glutamate (EC 1.4.4.13), and ammonia to L-glutamine (EC 6.3.1.2) were downregulated as well as the remaining genes in the whole nitrogen metabolism with high expression levels under coupled nitrogen and phosphorus limitation. Concomitantly, several genes were significantly upregulated under single nitrogen limitation, indicating that single nitrogen limitation might promote transcription. Four pathways harbored nitrogen metabolisms that include glyoxylate metabolism, arginine synthesis, citrate cycle, and glutamate metabolism. Among these pathways, syntheses of the arginine and citrate cycle contained a series of intermediates and were facilitated under nitrogen and phosphorus limitation.

**FIGURE 4:**
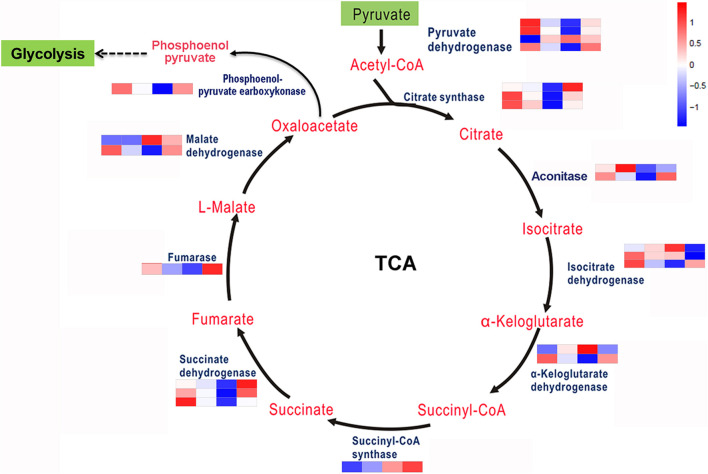
Key enzymes encoded by the DEGs involved in the carbohydrate metabolism are enriched by KEGG analysis.

The TCA cycle is an important aerobic pathway for the final steps of the oxidation of carbohydrates and FAs. A total of 22 DEGs encodings 10 enzymes were identified for the different steps in carbohydrate metabolism. Except for pyruvate dehydrogenase, citrate synthases, isocitrate dehydrogenase, and succinate dehydrogenase, which are encoded by 4, 3, 3, and 3 genes separately, the other enzymes are encoded by single or two genes (Figure 4). At the transcriptional level, aside from several genes encoding aconitase, isocitrate dehydrogenase and α-ketoglutarate dehydrogenase involved in this pathway were downregulated; all of the other enzyme-encoding genes involved in this pathway were upregulated under coupled nitrogen and phosphorus limitation. Conversely, the encoding genes of the majority of enzymes involved in the TCA cycle were identified as significantly downregulated under single nitrogen limitation, such as citrate synthases, aconitase, and succinate dehydrogenase. In summary, the TCA cycle represents one of the vital processes involved in FA formation during promoting and inhibiting carbon flux among the amino acids under nitrogen and phosphorus limitation.

### Expression Analysis of Linoleic Acid Biosynthesis Genes Under Nitrogen and Phosphorus Limitation

**FIGURE 5:**
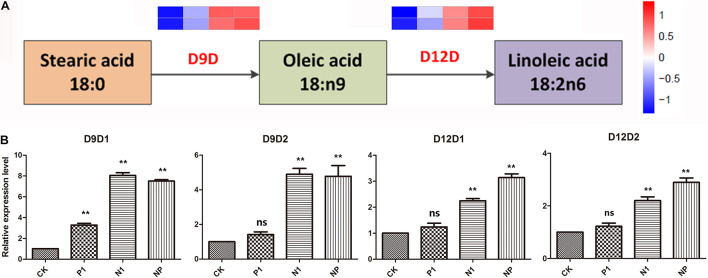
DEGs are involved in the linoleic acid biosynthesis pathways and the changes of C18 fatty acid in response to different nitrogen and phosphorus limitation. **(A)** The expression pattern of DEGs involved in the linoleic acid biosynthesis pathways. **(B)** The relative expression levels of four genes in response to different nitrogen and phosphorus limitation. The bars represent the average (±SE) of biological repeats. Asterisks indicate statistically significant differences between groups (Student’s *t*-test): **p < 0.01, ns: no significant difference. CK: the control; P1: the single P limitation (P-); N1: the single N limitation (N-); NP: N deep limitation and P limitation (N- -P-).

According to the KEGG pathway analysis, four genes involved in *A. oryzae* linoleic acid biosynthesis were identified. They encoded delta 9 fatty acid desaturases (D9D) and delta 12 fatty acid desaturases (D12D), which were rate-limiting enzymes in the biosynthesis of UFA and PUFAs, respectively. Analysis of gene expression levels for D9D and D12D revealed that the four genes involved in linoleic acid biosynthesis were upregulated in response to nitrogen and phosphorus limitation, indicating that UFA synthesis was enhanced in *A. oryzae* cells in response to different levels of nitrogen and phosphorus limitation (Figure 5A). Considering these four genes with remarkable expression levels in the single nitrogen and coupled limitation, the activity of D9D and D12D was enhanced at periods corresponding to linoleic acid production, suggesting a close correlation between nitrogen and phosphorus limitation and transcriptional regulation. This result was further supported by the relative expression levels of D9D1, D9D2, D12D1, and D12D2 genes (Figure 5B). Furthermore, in combination with the analysis resulted from the KEGG pathway, it was assumed that the conversion of stearic acid to linoleic acid through an intermediate (oleic acid) in *A. oryzae* cells might be a mechanism underlying the response to nitrogen and phosphorus limitation.

## Discussion

*Aspergillus oryzae* is an industrially oleaginous fungus that is previously reported to contain a high amount of lipid intracellularly (Meng et al., 2009). Although the pathway and related biosynthesis mechanisms of FA have been well documented, the dependency of this generally regarded as safe (GRAS) strain activity on nutrient availability in a medium is only partially understood. To achieve more insights on *A. oryzae* adaptations to nutrient-limiting conditions, precise physiological knowledge is needed. Consequently, we developed an experimental system assessing the effects of nutrient stresses on FA contents in *A. oryzae*. In this study, it was observed that biomass growths and spore density of *A. oryzae* in nitrogen-sufficient media (control) were significantly higher than nitrogen and phosphorus limited media and decreased with the decrease in nitrogen concentration (Figures 1A,B). Obviously, the effect of nitrogen limitation on the dry biomass and spore density of *A. oryzae* was beyond that of phosphorus limitation. The phenotype of *A. oryzae* strains under the condition of control and distinct N/P limitation validated this conclusion again (Figures 1C–H). It is generally accepted that proteins and other nitrogen-rich compounds are broken and utilized as nitrogen cell reservoirs to support time-restricted growth processes when microorganisms are cultured in a nitrogen-limited medium (Lv et al., 2010; Ördög et al., 2012). Therefore, it may explain the decrease in biomass content that occurred in nitrogen-limited treatments. Simultaneously, it was also observed that fungal growth (biomass and spore density) in the media with the phosphorus limitation were slightly (biomass) and substantially (spores density) lower than in the media with the moderate nitrogen-sufficient source concentrations (control) (Figures 1A,B). This result was consistent with a previously performed study by Dzurendova et al. (2020b), the use of low inorganic phosphorus sources (KH_2_PO_4_ and Na_2_HPO_4_) which play a buffering role in the growth media, and their low levels led to the decrease in pH. Low pH as a consequence of the low phosphorus source, thus, negatively affected the growth of oleaginous *Mucoromycota* fungi (Dzurendova et al., 2020b). KH_2_PO_4_ as inorganic phosphate salts utilized in the present study might function analogously. In addition, nitrogen and phosphorus limitation also inhibited the production of TFAs in *A. oryzae*, and the contents of palmitic acid, stearic acid, arachidic acid, and 2-decyltetradecanoic acid were decreased to a varying degree (Table 2). Since proteins and other nitrogen-rich compounds are broken, low pH and the synthesis of a set of phosphorylated molecules that involve the lipid accumulation process could be inhibited under the low phosphorus source availability (Dzurendova et al., 2020a). On the contrary, these four FAs, the contents of oleic acid, linoleic acid, and γ-linolenic acid were increased when strains were grown in the presence of nitrogen- and phosphorus-limited media. Additionally, it is noteworthy that the contents of these three FAs on P-medium were below that of the other media (Table 2). These results are in accordance with previous literature showing that a low phosphorus source amount resulted in the increment of the relative amount of the UFAs (γ-linolenic and linolenic) accompanied by the decrease in the amount of SFAs (oleic and stearic) in *Mucoraceae* fungi, except *Rhizopus stolonifer*. Moreover, the opposite effect was observed in *Mortierellaceae* fungi, where the amount of UFAs, specifically arachidonic acid, was reduced under low-phosphorus conditions (Dzurendova et al., 2020b). Similarly, in the finding of Hu et al. (2018), the proportion of TFAs and the concentration in the medium of linoleic acid, were greatly improved by a maximum of 1.6- and 1.1-fold, separately, under conditions of nitrogen or phosphorus deprivation compared with nitrogen and phosphorus sufficiency. The optimal conditions for the production of LC-PUFAs were also obtained by nitrogen or phosphorus stress (Hu et al., 2018).

Eukaryotic cells reprogram the expression of genes that are essential for metabolic pathways, accumulation of metabolic products, and adaption to the changing environments. The next-generation sequencing technologies have facilitated the research of the regulatory mechanisms of developmental events and metabolite biosynthesis on several filamentous fungi. In our previous study, RNA-seq was applied to study the transcriptome of *A. oryzae*, which displayed the dynamic changes of DEGs during *A. oryzae* growth (He et al., 2018a). Here, the same method was employed to study the transcriptome of *A. oryzae* under the condition of nitrogen and phosphorus limitation (Table 3). The results showed that the nutrient stress of nitrogen and phosphorus limitation led to a substantial transcriptional regulation of *A. oryzae* cells. DEGs (544) were identified on the condition of phosphorus limitation, the lowest among all treatments, among which, 304 were upregulated genes and 240 were downregulated genes (Figure 2A). Meanwhile, the number of DEGs reached a peak under the condition of both nitrogen and phosphorus limitation, which indicates that the response of *A. oryzae* to nitrogen and phosphorus limitation may be strong and different (Figure 2B). Furthermore, multiple pathways were altered in *A. oryzae* across the nutrient stress, nitrogen metabolism, and citrate cycle. The biological process of the nitrogen cycle is a complex interplay among many microorganisms catalyzing different reactions, where nitrogen is found in various oxidation states ranging from +5 in nitrate to −3 in ammonia. The high expression levels of most DEGs in whole nitrogen metabolism under coupled nitrogen and phosphorus limitation, indirectly illustrate the strong response of *A. oryzae* to nutrient stress (Figure 3). In contrast to the situation observed on that of nitrogen metabolism, the majority of enzyme-encoding genes involved in carbohydrate metabolism were significantly up/downregulated under coupled nitrogen and phosphorus limitation and single nitrogen limitation (Figure 4). These results indicated that the carbohydrate and nitrogen metabolism may also play a positive role in responding to nutrient stress of *A. oryzae*.

A remarkable increase was detected in fatty acid unsaturation, and a high proportion (approximately 12%) of stearic acid was converted into linoleic acid in *A. oryzae* cells on the N- -P-nutrient-limited group compared with the control (Table 2 and Supplementary Figure 1). Consistently, an increase in the unsaturation degree of FAs might counteract the elevated membrane fluidity caused by low phosphorus sources and maintain membrane stability in *Mucoraceae* fungi (Dzurendova et al., 2020b). Generally, the degree of FA unsaturation is significantly associated with membrane fluidity, and the upregulation of unsaturation is a critical strategy to maintain membranes in an appropriate fluid state (Srivastava et al., 2014). The overwhelming majority of cell membrane lipid moieties consist of UFAs that are synthesized from SFAs through specific enzymes (Tasaka et al., 1996; Weber et al., 2001). It was reported that UFA oleic acid was incorporated into the lipid membrane to compensate attenuation in membrane fluidity that counteracts the fluidizing effect of ethanol in the yeast *Saccharomyces cerevisiae* (You et al., 2003). Furthermore, it is worth noting that the biomass growths and spore density of *A. oryzae* might have a negative correlation with the degree of FA unsaturation (Figures 1A,B and Table 2) under the nutrient-limited treatments. Both of them were decreased and increased with the decrease in nitrogen concentration, respectively, illustrating the notable effect of nitrogen concentration on the growth and metabolic production of *A. oryzae*. Similarly, an increase in fatty acid unsaturation was observed under high ethanol conditions, while the biomass of *A. oryzae* strain was lower than that of the control (Ma et al., 2019). It is assumed that nitrogen nutrient limitation and specific ethanol stress may have the same effect on the development and FA biological synthesis of *A. oryzae*. D9D and D12D encountered a significant upregulation in transcription levels (Figure 5A), which indicates that nitrogen and phosphorus nutrient stress led to an induction of expression of fatty acid desaturases for fatty acid desaturation. It illustrated a tight correlation between transcriptional regulation and metabolite changes in lipid metabolism responding to nutrient stress. Analogously, the induced expression of fatty acid desaturases of *A. oryzae* cells was also observed under the condition of saline, ethanol, and oxidative stresses, in which the pathway of FA biosynthesis becomes active (He et al., 2018b; Ma et al., 2019; Shao et al., 2019).

In summary, we successfully characterized the transcriptomic profiles, which uncovered new aspects of the dynamic changes in *A. oryzae* mycelium growth and conidia formation over time under nitrogen and phosphorus nutrient stress. The improvement of linoleic acid synthesis was characterized, and the understanding of the mechanisms involved in the nutrient stress of *A. oryzae* was also improved. Transcriptomic analysis showed high expression levels of the genes related to linoleic acid synthesis, resulting in a corresponding intracellular accumulation of linoleic acid. Our study provides a global transcriptome characterization of the nitrogen and phosphorus limitation adaptation process in *A. oryzae*. It also laid a solid foundation for further investigation to explore the relationship of UFA accumulation and nitrogen and phosphorus limitation in *A. oryzae*.

## Data Availability Statement

The datasets presented in this study can be found in online repositories. The names of the repository/repositories and accession number(s) can be found below: NCBI/SRA database (https://www.ncbi.nlm.nih.gov/sra, Bioproject: PRJNA748192; Biosamples: SAMN20309416-SAMN20309419).

## Author Contributions

GL conceptualized the study and wrote and prepared the original draft. YX conceptualized the study. XC and YT were in charge of the project administration. BZ supervised the study and acquired the funding. JH supervised the study. BH wrote reviewed, and edited the manuscript, and acquired the funding. All authors contributed to the article and approved the submitted version.

## Conflict of Interest

The authors declare that the research was conducted in the absence of any commercial or financial relationships that could be construed as a potential conflict of interest.

## Publisher’s Note

All claims expressed in this article are solely those of the authors and do not necessarily represent those of their affiliated organizations, or those of the publisher, the editors and the reviewers. Any product that may be evaluated in this article, or claim that may be made by its manufacturer, is not guaranteed or endorsed by the publisher.

## Footnotes

1
http://bowtie-bio.sourceforge.net/bowtie2

2
https://www.ncbi.nlm.nih.gov/sra

## Supplementary Material

Supplementary Figure 1 Intracellular content of FA under nitrogen and phosphorus limitation and the control according to the saturability of FAs. CK, N-, P-, N- -, N- P-, and N- -P- represent the control and distinct N/P-limited groups, respectively.

Supplementary Table 1 The dried biomass of distinct nutrient-limited groups. CK, N-, P-, N- -, N- P-, and N- -P- represent the control and distinct N/P-limited groups, respectively.
