# Supplementary figures and images for: Effects of Nitrogen and Phosphorus Limitation on Fatty Acid Contents in Aspergillus oryzae
Source: Front Microbiol. 2021 Oct 21;12:739569. doi: 10.3389/fmicb.2021.739569 (PMC8566876; doi:10.3389/fmicb.2021.739569)

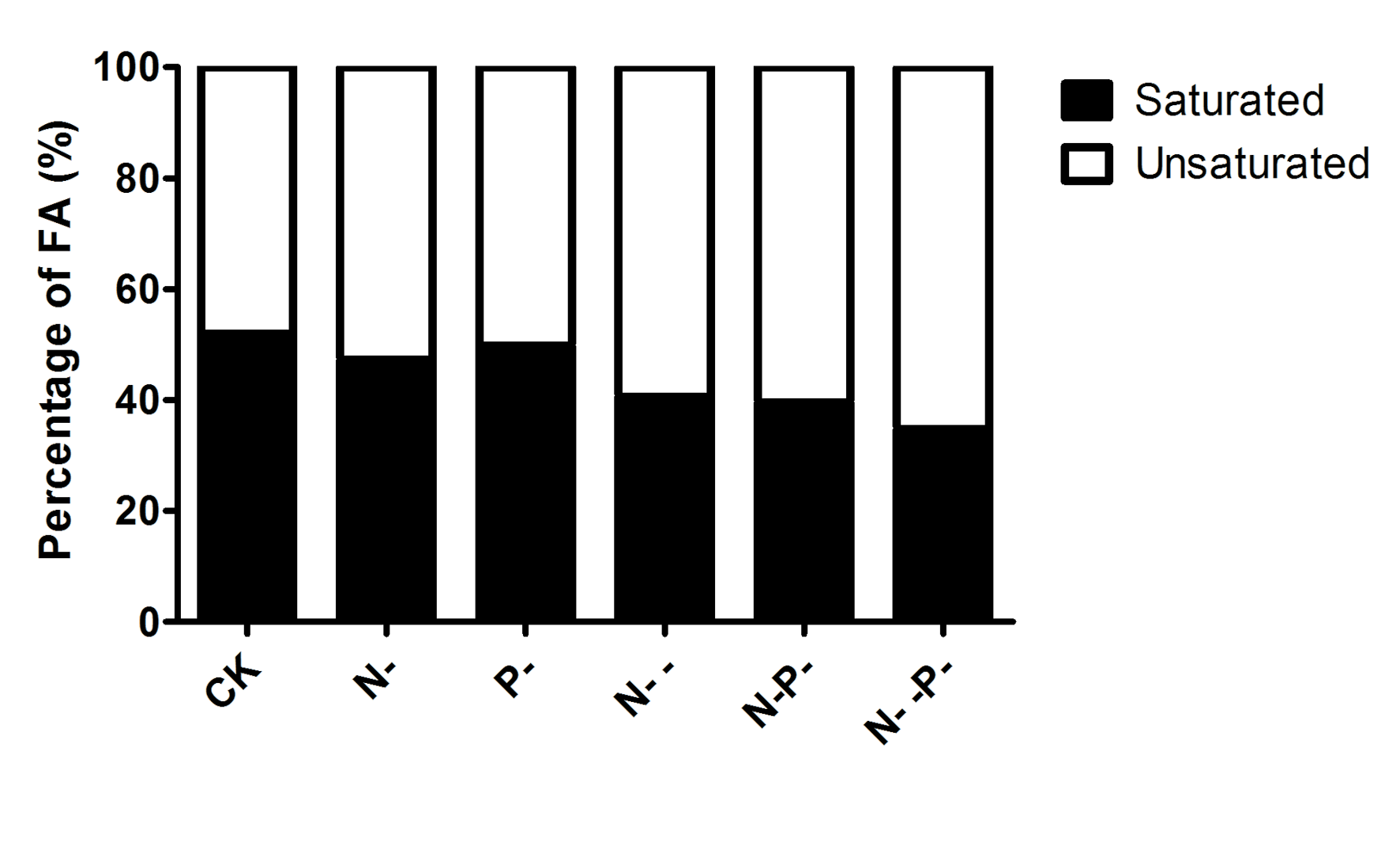

Supplement: Supplementary Figure 1 — Intracellular content of FA under nitrogen and phosphorus limitation and the control according to the saturability of FAs. CK, N-, P-, N- -, N- P-, and N- -P- represent the control and distinct N/P-limited groups, respectively. [file Image_1.TIF]
